# Clinicopathological Features and Prognostic Implication of Gastric Carcinoma with Lymphoid Stroma

**DOI:** 10.1155/2020/6628412

**Published:** 2020-12-03

**Authors:** Jung-Soo Pyo, Nae Yu Kim, Byoung Kwan Son, Hyo Young Lee, Il Hwan Oh, Kwang Hyun Chung

**Affiliations:** ^1^Department of Pathology, Daejeon Eulji University Hospital, Eulji University School of Medicine, Daejeon, Republic of Korea; ^2^Department of Internal Medicine, Daejeon Eulji University Hospital, Eulji University School of Medicine, Daejeon, Republic of Korea; ^3^Department of Internal Medicine, Nowon Eulji University Hospital, Eulji University School of Medicine, Seoul, Republic of Korea

## Abstract

**Methods:**

This study included 34 eligible studies and 1757 GCLSs. The clinicopathologic characteristics of GCLS were investigated from eligible studies, and the meta-analysis was performed. In addition, we compared the survival rates between GCLS and non-GCLS.

**Results:**

The estimated rate of GCLS was 0.062 (95% confidence interval (CI) 0.040-0.097). GCLS was significantly correlated with the diffuse type of Lauren's classification, proximal tumor location, less-frequent lymphatic invasion, and lower pTNM stage. However, there was no significant difference in age, sex, tumor differentiation, vascular invasion, perineural invasion, pT stage, lymph node metastasis, and distant metastasis between GCLS and non-GCLS patients. EBV positive rates in GCLS and non-GCLS patients were 0.723 (95% CI 0.643-0.791) and 0.064 (95% CI 0.039-0.103), respectively. HER2 expression in GCLS was significantly lower than that in non-GCLS. GCLS patients had a more favorable prognosis than that of non-GCLS patients (hazard ratio 0.500, 95% CI 0.305-0.821).

**Conclusion:**

GCLS comprised 6.2% of overall GC and more frequent in the proximal portion of the stomach. Since GCLS was associated with better prognosis, the histologic finding can be useful for predicting the patient's prognosis.

## 1. Introduction

Gastric carcinoma (GC) includes various subtypes, such as tubular adenocarcinoma, poorly cohesive carcinoma, and GC with lymphoid stroma (GCLS) [1]. Among these subtypes, GCLS accounts for 1-7% of overall GC [1]. According to the WHO classification, medullary and lymphoepithelioma-like carcinomas are synonyms for GCLS [1]. In GCLS, tumor cells have irregular sheets, trabeculae, ill-defined tubules, or syncytial pattern [1]. In addition, the characteristic histology is a prominent lymphocytic infiltrate with intraepithelial lymphocytes [2–35]. Although GCLS usually has a well-defined tumor border, small clusters within prominent intratumoral lymphocytes can have infiltrative borders [36]. GCLS is known to correlate with the male sex, proximal tumor location, and Epstein-Barr virus (EBV) positivity. However, some clinicopathologic features of GCLS vary [2–35]. In addition, histologic features of GCLS, which has a low prevalence, may overlap with that of poorly differentiated adenocarcinoma. These factors can affect variable clinicopathologic features between reports. EBV positivity was frequently found in GCLS. However, detailed clinicopathologic features with respect to EBV positivity are unclear. We investigated the prevalence of GCLS in overall GC through a meta-analysis. In addition, the clinicopathologic features and prognosis between GCLS and non-GCLS were compared. In addition, subgroup analysis based on EBV positivity was performed in predicting the prognosis of GCLS.

## 2. Materials and Methods

### 2.1. Published Study Search and Selection Criteria

Relevant articles were obtained by searching the PubMed database through April 30, 2020. We used the following keywords: “stomach or gastric” and “gastric carcinoma with lymphoid stroma or medullary carcinoma or lymphoepithelioma-like carcinoma.” The titles and abstracts of all searched articles were screened for inclusion and exclusion. Included articles should have information on clinicopathological characteristics or prognosis in GCLS. However, nonoriginal articles, such as case reports and review articles, were excluded. In addition, those not written in English were not included in the present study. This protocol was reviewed and approved by the Institutional Review Board of Eulji University Hospital (EMC 2020-09-007).

### 2.2. Data Extraction

Data extracted from 34 eligible studies [2–35] included the author's information, study location, number of patients analyzed, the prevalence and the clinicopathological characteristics of GCLS, the correlation with various markers, and overall survival rates of GCLS. For the quantitative aggregation of the survival results, the correlation between GCLS and survival was analyzed according to the hazard ratio (HR) using one of three methods. In studies that did not record the HRs or confidence intervals (CIs), we calculated these variables from the data using the HR point estimate, the log-rank statistic or its *P* value, and the O-E statistic (the difference between the number of observed and expected events) or its variance. If these data were unavailable, the HR was estimated using the total number of events, number of patients at risk in each group, and the log-rank statistic or its *P* value. Finally, if the only useful data were in the form of graphical representations of survival distributions, survival rates were extracted at specified times to reconstruct the HR estimate and its variance under the assumption that patients were censored at a constant rate during the time intervals [37]. The published survival curves were read independently by two authors in order to reduce variability. The HRs were then combined using Peto's method [38]. Data associated with survival were extracted after a 60-month follow-up period. All data were obtained by two independent authors.

### 2.3. Statistical Analyses

The meta-analysis was performed using the Comprehensive Meta-Analysis software package (Biostat, Englewood, NJ, USA). The prevalence of GCLSs among GC was investigated. Subgroup analyses based on the depth of the tumor, was performed. The clinicopathological characteristics of GCLS, such as age, sex, size, tumor differentiation, lymphovascular invasion, and pTNM stages, were compared with those of non-GCLS. EBV positivity from EBER in situ hybridization between GCLS and non-GCLS was compared. In addition, the differences of PD-L1, HER2, and p53 immunohistochemical expressions between GCLS and non-GCLS were investigated. Heterogeneity between the studies was checked by the *Q* and *I*^2^ statistics and expressed as *P* values. Additionally, sensitivity analysis was conducted to assess the heterogeneity of eligible studies and the impact of each study on the combined effects. Eligible studies included various populations having different tumor subtypes, tumor stages, and treatments. In addition, although the molecular and immunohistochemical tests were qualified, the methods were different between laboratories. Thus, in interpretations for estimated results, a random-effect model rather than a fixed-effect model was used. To assess publication bias, Begg's funnel plot and Egger's test were used; if it was significant, the fail-safe *N* and trim-fill tests were additionally used to confirm the degree of publication bias. The results were considered statistically significant at *P* < 0.05.

## 3. Results

### 3.1. Selection and Characteristics of the Studies

**Figure 1:**
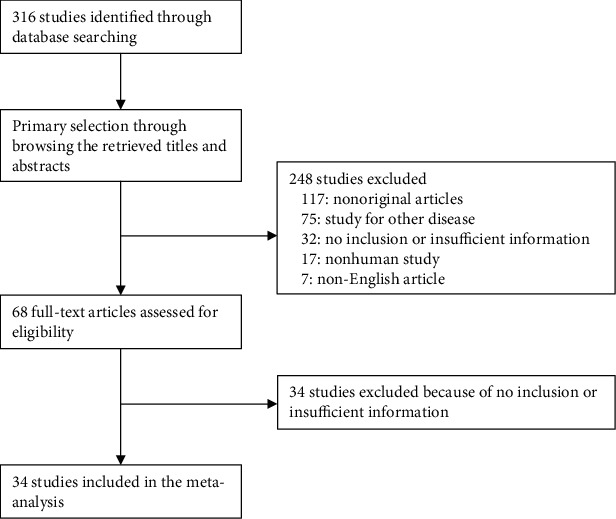
Flow chart of study search and selection methods.

From the primary search using the PubMed database, we found 316 relevant articles. In screening and reviewing, we excluded 117 because they were not original. Sixty-six articles had no information or insufficient information for a meta-analysis. Among the remaining articles, 489 reports were excluded for the following reasons: articles reporting other diseases (*n* = 75), nonhuman studies (*n* = 17), and a language other than English (*n* = 7) (Figure 1). Finally, 34 eligible articles were included in the meta-analysis (Table 1). These studies included 1757 GCLS and 14,926 non-GCLS patients.

### 3.2. Prevalence of GCLSs

The estimated prevalence rate of GCLS among GCs was 0.062 (95% CI 0.040-0.097) (Table 2). In early GC (EGC) and advanced GC (AGC), GCLSs made up 0.054 (95% CI 0.022-0.129) and 0.136 (95% CI 0.119-0.155), respectively. There was no significant difference in the prevalence of GCLS between EGC and AGC (*P* = 0.392 in a metaregression test). In addition, in sensitivity tests, there was no significant impact of each study on estimated prevalence rate.

### 3.3. Comparisons of Clinicopathological Characteristics between GCLS and Non-GCLSs

Next, we compared the clinicopathological characteristics between GCLS and non-GCLS patients. Statistical significances was found in the tumor size, Lauren's classification, tumor location, lymphatic invasion, and pTNM stage using the metaregression tests (Table 3). The tumor sizes were 3.275 cm (95% CI 2.521-4.029 cm) and 4.636 cm (95% CI 3.786-5.486 cm), respectively. GCLSs were frequently found in the diffuse type of Lauren's classification. Tumors occurring in the proximal 1/3 of the stomach comprised 24.9% and 14.8% of GCLS and non-GCLS, respectively. The lymphatic invasion was less frequent in GCLS than in non-GCLS. Stage I and II GCs occurred in 0.750 (95% CI 0.599-0.858) and 0.500 (95% CI 0.317-0.683) of GCLS and non-GCLS, respectively. However, there were no significant differences in the age, sex, tumor differentiation, vascular invasion, perineural invasion, pT stage, and lymph node metastasis.

EBV positivity was found in 72.3% of GCLSs and 6.4% of non-GCLSs, respectively (Table 4). There was a significant difference in EBV positivity between GCLSs and non-GCLSs (*P* < 0.001 in the metaregression test). Microsatellite instability was found in 5.9% of GCLSs. PD-L1 immunohistochemical expression rates in GCLS occurred in 0.677 (95% CI 0.497-0.817) and 0.742 (95% CI 0.563-0.865) in tumor and immune cells, respectively. HER2 immunohistochemical expression rates were 0.026 (95% CI 0.005-0.120) and 0.632 (95% CI 0.403-0.813) in GCLS and non-GCLS, respectively.

### 3.4. Comparison of Prognosis between GCLS and Non-GCLSs

Patients with GCLS had a better overall survival than those with non-GCLS (HR 0.500, 95% CI 0.305-0.821; Table 5). However, there were no significant differences in overall survival rates between GCLS and non-GCLS in EGC. In EBV-associated GC, patients with GCLS had better overall survivals than those with non-GCLS (HR 0.090, 95% CI 0.025-0.319).

## 4. Discussion

The present study is the first meta-analysis, to the best of our knowledge, to elucidate the clinicopathological characteristics of GCLS. There were four significant findings in this study. First, the estimated prevalence of GCLS was 6.2% of all GCs. Second, GCLS had a smaller tumor size and higher frequency of the diffuse type of Lauren's classification, occurred in the proximal portion of the stomach, had lymphatic invasion, and had lower pTNM stages compared to non-GCLS. Third, GCLS significantly correlated with EBV positivity in situ hybridization. Fourth, GCLS had a favorable prognosis than non-GCLS.

GCLSs comprise 1-7% of all GCs [1]. In our eligible studies, the prevalence of GCLS ranged from 1.7% to 16.5% [16, 27]. In a meta-analysis, the estimated rate of GCLS was 0.062 (95% CI 0.040-0.097). The prevalence rates were 0.054 and 0.136 in EGC and AGC, respectively. However, there was no significant difference in GCLS rates between EGC and AGC (*P* = 0.392 in the metaregression test). As shown in Table 3, the estimated rates of pT1/T2 were 0.592 and 0.428 in GCLS and non-GCLS, respectively. In addition, there was no statistical difference in pT1/T2 rates between GCLS and non-GCLS. According to the WHO classification, GCLS significantly correlated with the proximal stomach/remnant stomach and male sex, as seen in our results [1]. However, because the incidence of GCLS is lower than that of other subtypes, those clinicopathological features can be controversial between studies. In addition, GCLS significantly correlated with a smaller tumor size, diffuse type of Lauren's classification, a lower frequency of lymphatic invasion, and a lower pTNM stage compared to non-GCLS. From our results, the estimated rates of lymphatic and vascular invasions were 25.9% and 14.6%, respectively. Lim et al. reported that there was no lymphovascular invasion of GCLS in EGC [17]. However, we could not perform the subgroup analysis for lymphovascular invasion based on the pT stage due to insufficient information. To obtain detailed information, a cumulative study is needed.

Lim et al. reported treatment results of endoscopic resection for the early stage of GCLS (pT1/2) [17]. Histologic differentiation may be important in deciding the treatment modality. However, among 40 GCLSs, only 10% of cases were diagnosed as GCLS on the pretreatment biopsy. The remaining cases were diagnosed as differentiated adenocarcinomas (60%) and undifferentiated adenocarcinomas (20%) [17]. On the pretreatment biopsy, it is difficult to assess whether a poorly differentiated tumor is a GCLS. In our result, GCLS had a low frequency of lymphatic invasion than non-GCLS. In addition, although there was no statistical difference, GCLS showed less-frequent vascular invasion and lymph node metastasis and lower pT1/T2 than non-GCLS. This result suggests that histologic findings of GCLS may not be a contraindication for endoscopic resections, regardless of pathologically confirmed diagnosis. Lim et al. reported that the rate of en bloc resection with endoscopic resection was 97.5% in GCLS at early stages [17]. In other reports, the complete resection rate was 60-80% in undifferentiated EGCs [39–41]. The histologic characteristics of GCLS, which is embedded by peritumoral lymphocytes, may be affected by this higher complete resection rate. In the pretreatment diagnosis, it is necessary to consider a GCLS when differentiating from poorly/undifferentiated adenocarcinoma.

Medullary carcinoma is also described in colorectal and breast cancers. The characteristic histologic finding of these carcinomas is poorly differentiated tumor cells with peritumoral lymphocytic infiltration. However, diagnostic criteria are slightly different between tumors. In breast cancer, medullary carcinoma is defined as (1) sheets of cells with indistinct cell borders (syncytial growth) in greater than 75% of the tumor, (2) sharply circumscribed and pushing borders, and (3) moderate to poor differentiation [42]. In colorectal cancers, the malignant, well-circumscribed neoplasm has a solid growth pattern (no gland formation) and pushing border. We previously reported the clinicopathologic characteristics and prognosis of colorectal medullary carcinomas through a meta-analysis [43]. In colorectal cancers, patients with medullary carcinoma had a significantly better overall survival rate compared to patients with poorly differentiated and undifferentiated adenocarcinoma [43]. In the present study, GCLS had a better prognosis than non-GCLS. However, in the subgroup analysis on tumor depth (EGC vs. AGC), a different result was obtained. There was no significant difference in survival rate between GCLS and non-GCLS in EGCs. However, in AGCs, the prognosis was better in GCLS than that in non-GCLS. The estimated rate of pT1/T2 was 59.2% in all GCLSs. This result confirmed that the better prognosis of GCLS was not caused by a lower pT1/T2 rate compared to non-GCLS. In colorectal cancer, medullary carcinoma had a better prognosis than poorly differentiated and undifferentiated adenocarcinoma. As described above, GCLS significantly correlated with EBV positivity. We checked the difference in survival between EBV positivity and negativity in GCLS. However, there was no significant difference in prognosis between the two groups.

The characteristic finding of GCLS is peritumoral and tumor-infiltrating lymphocytes. In addition, cell nests of GCLS can be embedded in prominent lymphocytic infiltrates. Because studies against the immunotherapeutic effects of various cancers are recently increasing, the correlation between TILs and PD-L1 expression is useful to understand the treatment in GCLS. Because EBV positivity of GCLS was high, molecular characteristics of GCLS and EBVaGC may be overlapping. PD-L1 gene amplification was elevated in EBV-associated GCs [44]. In the previous meta-analysis, the PD-L1 expression rate of tumor cells was 47.0% in GCs [45]. In the present study, tumor cells of GCLS were found in 67.7% of GCLSs. The PD-L1 expression rates were higher in GCLSs than that in overall cases. Immune cells showed PD-L1 expression in 74.2% of GCLS. Although PD-L1 expressions between GCLS and non-GCLS could not be compared, high PD-L1 expression of GCLS is meaningful. Because the implication of tumor-infiltrating lymphocytes is not clear in GC, further evaluation in GCLS will be needed.

In GCLS, EBV positivity varied from 22.6% to 100% by reports [9, 27]. We found higher EBV positivity in GCLS than in non-GCLS (72.3% vs. 6.4%; *P* < 0.001 in the metaregression test). The positive rates of EBV in non-GCLS varied from 0% to 19% among eligible studies [5, 32]. However, because the EBV positive rate was not 100%, the comparison of clinicopathological characteristics between EBV positive (EBVaGCLS) and negative GCLSs (non-EBVaGCLS) can be useful in understanding GCLS. Chang et al. reported that EBVaGCLS significantly correlated with the male sex, the middle third of the stomach, and the diffuse type of Lauren's classification compared to non-EBVaGCLS [2]. Min et al. reported that non-EBVaGCLS correlated with older age, female, advanced T stage, and advanced pTNM [20]. Previous studies have reported that EBV positivity significantly correlated with sex and tumor location [21,25,52]. Also, GCLS significantly correlated with male patients and proximal tumor location. These characteristics of GCLS overlapped with EBVaGC. The cause may be a high EBV positive rate of GCLS. In addition, GCLS significantly correlated with less-frequent lymphatic invasion and lower pTNM stage. Min et al. reported that EBV positive cases showed a better overall survival rate than EBV negative cases in GCLS [20]. However, there was no significant difference in survival rate between EBVaGCLS and non-EBVaGCLS in our study. We additionally evaluated survival rates between GCLS and non-GCLS in EBVaGCs. Patients with GCLS showed a favorable prognosis than those with non-GCLS in EBVaGCs (HR 0.090, 95% CI 0.025-0.319).

This study has some limitations. First, the analysis of MSI status in GCLS could not be performed due to insufficient information. Setia et al. reported that one case out of 17 GCLS showed a microsatellite instable (MSI) status [29]. Second, PD-L1 expression rates were only shown in GCLS, but not in non-GCLS. In included studies, there was no information for PD-L1 expression of non-GCLS. In conclusion, GCLS was found in 6.2% of overall GC. In addition, GCLS was significantly correlated with male patients and the proximal tumor location of the stomach. Because GCLS was associated with a better prognosis, the histologic finding can be useful for predicting the patient's prognosis.

## Figures and Tables

**Table 1:** Main characteristics of the eligible studies.

	Location	Patient's group	Number of patients	EBV positive rate∗		Location	Patient's group	Number of patients	EBV positive rate∗
Chang 2000	Korea	GCLS w/EBV+	30	66.7%	Lopes 2004	Brazil	GCLS	7	57.1%
		GCLS w/EBV-	15		Lu 2004	China	GCLS	17

Chang 2001	Korea	GCLS	14	50.0%			Non-GCLS	64	
		Non-GCLS	292	3.4%	Min 2016	Korea	GCLS	145	85.5%

Cheng 2015	China	GCLS w/EBV+	8		Mohri 2017	Japan	GCLS	15	66.7%
		Non-GCLS w/EBV +	45		Ohtani 2009	Japan	GCLS	24	91.7%

Cho 2003	Korea	GCLS	9	77.8%			Non-GCLS	35	5.7%
		Non-GCLS	21	19.0%	Osumi 2019	Japan	GCLS	80	75.0%

Cho 2004	Korea	GCLS	24	79.2%	Otsuji 2004	Japan	GCLS	31	
		Non-GCLS	23	8.7%			Non-GCLS w/PD	136	

Gonzalez 2017	USA	GCLS	13				Non-GCLS w/WD	799	

Hirai 2016	Japan	GCLS	23		Park 2015	Korea	GCLS	46	80.4%

Hissong 2018	USA	GCLS	31	22.6%			Non-GCLS	4236	6.5%

Huang 2013	Taiwan	GCLS in EGC	31		Ramos 2017	Brazil	GCLS	7	
		Non-GCLS in EGC	520				Non-GCLS	248	
		GCLS in AGC	191		Ribeiro 2017	Portugal	GCLS	3	100.0%
		Non-GCLS in AGC	1217		Selves 1996	France	GCLS	6	66.7%

Huang 2014	Taiwan	GCLS w/EBV+	18		Setia 2019	USA	GCLS	17	64.7%
		Non-GCLS w/EBV +	33		Shin 2017	Korea	GCLS	70	

Huh 2016	Korea	GCLS	41	72.2%			Non-GCLS	1626	
		Non-GCLS	3344		Song 2010	USA	GCLS w/EBV+	53	

Jing 1997	Japan	GCLS	8	37.5%			Non-GCLS w/EBV +	18	

Kang 2016	Korea	GCLS	60		Tobo 2013	Japan	GCLS	104	75.0%
		Non-GCLS	60				Non-GCLS	29	0.0%

Lim 2015	Korea	GCLS	274	86.1%	Wu 2000	Taiwan	GCLS w/EBV+	11	
		Non-GCLS	822				Non-GCLS w/EBV +	19	

Lim 2017	Korea	GCLS	241	89.2%			Non-GCLS w/EBV-	120	
		Non-GCLS	1219		Yanagi 2019	Japan	GCLS	43	60.5%

Lim 2018	Korea	GCLS	40	90.0%	Yuen 1994	Hong Kong	GCLS	7	28.6%

∗In gastric carcinoma with lymphoid stroma; EBV, Epstein-Barr virus; GCLS, gastric carcinoma with lymphoid stroma; w/, with; EGC, early gastric carcinoma; AGC, advanced gastric carcinoma; PD, poorly differentiated; WD, well differentiated.

**Table 2:** The estimated rates of gastric carcinoma with lymphoid stroma.

	Number of subsets	Fixed effect (95% CI)	Heterogeneity test (*P* value)	Random effect (95% CI)	Egger's test (*P* value)	Meta-regression test (*P* value)
GCLS rate	20	0.097 [0.092, 0.103]	<0.001	0.062 [0.040, 0.097]	0.023	0.392∗
EGC	5	0.087 [0.079, 0.095]	<0.001	0.054 [0.022, 0.129]	0.077	
AGC	1	0.136 [0.119, 0.155]	1.000	0.136 [0.119, 0.155]	—	

CI, confidence interval; GCLS, gastric carcinoma with lymphoid stroma; EGC, early gastric carcinoma; AGC, advanced gastric carcinoma ∗EGC vs. AGC.

**Table 3:** Clinicopathological significances of gastric carcinoma with lymphoid stroma.

	Number of subsets	Fixed effect (95% CI)	Heterogeneity test (*P* value)	Random effect (95% CI)	Egger's test (*P* value)	Meta-regression test (*P* value)
Age						
GCLS	13	57.892 [57.293, 58.491]	<0.001	58.182 [56.857, 59.507]	0.412	0.152
Non-GCLS	11	58.252 [58.059, 58.445]	<0.001	59.864 [58.410, 61.318]	0.164	

Male ratio						
GCLS	26	0.794 [0.772, 0.815]	<0.001	0.765 [0.712, 0.811]	0.064	0.052
Non-GCLS	18	0.686 [0.678, 0.694]	<0.001	0.709 [0.665, 0.751]	0.333	

Size (cm)						
GCLS	7	2.763 [2.618, 2.908]	<0.001	3.275 [2.521, 4.029]	0.181	0.032
Non-GCLS	7	2.908 [2.865, 2.951]	<0.001	4.636 [3.786, 5.486]	0.018	

Lauren's classification, diffuse type						
GCLS	10	0.554 [0.504, 0.602]	<0.001	0.577 [0.437, 0.705]	0.512	0.036
Non-GCLS	8	0.444 [0.433, 0.455]	<0.001	0.455 [0.397, 0.514]	0.949	

Tumor differentiation, poorly						
GCLS	8	0.407 [0.369, 0.446]	<0.001	0.710 [0.474, 0.870]	0.070	0.376
Non-GCLS6	6	0.618 [0.605, 0.630]	0.009	0.610 [0.574, 0.645]	0.859	

Tumor location, proximal 1/3						
GCLS	15	0.280 [0.254, 0.308]	<0.001	0.249 [0.190, 0.319]	0.223	<0.001
Non-GCLS	14	0.132 [0.126, 0.137]	<0.001	0.148 [0.123, 0.177]	0.375	

Lymphatic invasion						
GCLS	6	0.330 [0.253, 0.417]	<0.001	0.259 [0.113, 0.490]	0.369	0.024
Non-GCLS	7	0.381 [0.368, 0.395]	<0.001	0.516 [0.405, 0.625]	0.116	

Vascular invasion						
GCLS	7	0.257 [0.193, 0.334]	<0.001	0.146 [0.049, 0.361]	0.336	0.666
Non-GCLS	8	0.127 [0.117, 0.137]	<0.001	0.214 [0.120, 0.353]	0.099	

Perineural invasion						
GCLS	13	0.150 [0.122, 0.183]	<0.001	0.104 [0.056, 0.184]	0.170	0.335
Non-GCLS	9	0.129 [0.122, 0.137]	<0.001	0.158 [0.081, 0.284]	0.977	

pT stage, pT1/T2						
GCLS	14	0.624 [0.587, 0.660]	<0.001	0.592 [0.472, 0.702]	0.559	0.102
Non-GCLS	12	0.728 [0.716, 0.740]	<0.001	0.428 [0.301, 0.565]	0.005	

Lymph node metastasis						
GCLS	20	0.351 [0.322, 0.381]	<0.001	0.308 [0.217, 0.417]	0.368	0.100
Non-GCLS	15	0.324 [0.314, 0.333]	<0.001	0.462 [0.322, 0.608]	0.486	

Distant metastasis						
GCLS	2	0.197 [0.057, 0.499]	<0.001	0.197 [0.057, 0.499]	—	—

pTNM stage, I/II						
GCLS	13	0.696 [0.659, 0.731]	<0.001	0.750 [0.599, 0.858]	0.428	0.042
Non-GCLS	11	0.685 [0.673, 0.697]	<0.001	0.500 [0.317, 0.683]	0.240	

CI, confidence interval; GCLS, gastric carcinoma with lymphoid stroma.

**Table 4:** The estimated rates of various markers in gastric carcinoma with lymphoid stroma.

	Number of subsets	Fixed effect (95% CI)	Heterogeneity test (*P* value)	Random effect (95% CI)	Egger's test (*P* value)	Meta-regression test (*P* value)
EBV positivity						
GCLS	22	0.780 [0.754, 0.805]	<0.001	0.723 [0.643, 0.791]	0.029	<0.001
Non-GCLS	6	0.063 [0.052, 0.076]	0.067	0.064 [0.039, 0.103]	0.970	

Microsatellite instable						
GCLS	1	0.059 [0.008, 0.320]	1.000	0.059 [0.008, 0.320]	—	—

PD-L1 in tumor cells						
GCLS	1	0.677 [0.497, 0.817]	1.000	0.677 [0.497, 0.817]	—	—

PD-L1 in immune cells						
GCLS	1	0.742 [0.563, 0.865]	1.000	0.742 [0.563, 0.865]	—	—

HER2						
GCLS	3	0.026 [0.005, 0.120]	0.883	0.026 [0.005, 0.120]	0.089	<0.001
Non-GCLS	1	0.632 [0.403, 0.813]	1.000	0.632 [0.403, 0.813]	—	

p53						
GCLS	5	0.368 [0.253, 0.499]	<0.001	0.386 [0.140, 0.707]	0.854	0.554
Non-GCLS	2	0.494 [0.388, 0.600]	0.401	0.494 [0.388, 0.600]	—	

CI, confidence interval; EBV, Epstein-Barr virus.

**Table 5:** The prognostic implications of gastric carcinoma with lymphoid stroma.

	Number of subsets	Fixed effect (95% CI)	Heterogeneity test (*P* value)	Random effect (95% CI)	Egger's test (*P* value)
GCLS vs. non-GCLS					
Overall	6	0.854 [0.767, 0.951]	<0.001	0.500 [0.305, 0.821]	0.023
EGC	1	1.022 [0.788, 1.325]	1.000	1.022 [0.788, 1.325]	—
AGC	1	0.863 [0.765, 0.972]	1.000	0.863 [0.765, 0.972]	—
EBVaGC	2	0.090 [0.025, 0.319]	0.884	0.090 [0.025, 0.319]	—
EBV+ vs. EBV- in GCLS	2	0.573 [0.305, 1.076]	0.147	0.477 [0.160, 1.425]	—

CI, confidence interval; GCLS, gastric carcinoma with lymphoid stroma; EGC, early gastric carcinoma; AGC, advanced gastric carcinoma; EBV, Epstein-Barr virus.

## Data Availability

The data supporting this meta-analysis are from previously reported studies and datasets, which have been cited. There was the processed data from the corresponding author upon request.
